# Effect of Inlet and Outlet Flow Conditions on Natural Gas Parameters in Supersonic Separation Process

**DOI:** 10.1371/journal.pone.0110313

**Published:** 2014-10-22

**Authors:** Yan Yang, Chuang Wen, Shuli Wang, Yuqing Feng

**Affiliations:** 1 Jiangsu Key Laboratory of Oil-Gas Storage and Transportation Technology, Changzhou University, Changzhou, Jiangsu Province, China; 2 Computational Informatics, Commonwealth Scientific and Industrial Research Organization, Melbourne, The State of Victoria, Australia; Northwestern Polytechnical University, China

## Abstract

A supersonic separator has been introduced to remove water vapour from natural gas. The mechanisms of the upstream and downstream influences are not well understood for various flow conditions from the wellhead and the back pipelines. We used a computational model to investigate the effect of the inlet and outlet flow conditions on the supersonic separation process. We found that the shock wave was sensitive to the inlet or back pressure compared to the inlet temperature. The shock position shifted forward with a higher inlet or back pressure. It indicated that an increasing inlet pressure declined the pressure recovery capacity. Furthermore, the shock wave moved out of the diffuser when the ratio of the back pressure to the inlet one was greater than 0.75, in which the state of the low pressure and temperature was destroyed, resulting in the re-evaporation of the condensed liquids. Natural gas would be the subsonic flows in the whole supersonic separator, if the mass flow rate was less than the design value, and it could not reach the low pressure and temperature for the condensation and separation of the water vapor. These results suggested a guidance mechanism for natural gas supersonic separation in various flow conditions.

## Introduction

As the global economy rises, the demand for energy supply is increasing continuously in the last two decades. Natural gas plays a significant strategic role in the energy supply [1]. Natural gas is gaseous mixture, primarily composed of methane, ethane, propane and butane, with some heavier alkanes, carbon dioxide, hydrogen sulfide, nitrogen and a small amount of water vapor [2]. The presence of water vapor in natural gas increases the risk of the formation of gas hydrates with line plugging due to hydrate deposition on the pipe walls, results in corrosion combined with acid gases including carbon dioxide and hydrogen sulfide, and reduces the delivery capacity of the pipelines because of the collection of free water [3]. Consequently, the water vapor must be removed from natural gas early on.

At present, many conventional techniques are employed for the natural gas separation, such as absorption, adsorption, refrigeration, membranes and so on. A supersonic separator, as a novel technique, has been introduced to natural gas processing from the beginning of this century [4]–[6]. In essence, the supersonic separation technique causes refrigeration like the Joule-Thompson effect and Turbine expansion, both of which induce a low temperature for the condensation of water vapor. The supersonic separator mainly consists of a Laval nozzle, a swirl device and a diffuser.

Malyshkina [7], [8] obtained the distribution of gas dynamic parameters through a supersonic separator with a computational method, and a procedure was developed to predict the separation capability of water vapor and higher hydrocarbons from natural gas by using a supersonic separator determined by the initial parameters. Karimi and Abdi [9] studied the flow fields of natural gas in a Laval nozzle of 0.12 m long. But the working fluid was assumed to be a supercritical flow. The geometric construction and flow conditions are quite different from the actual flow states of natural gas in a supersonic separator for dehydration. Jiang et al. [10] employed the corrected Internally Consistent Classical Theory and Gyarmathy theory to modelling the nucleation and droplet growth of natural gas in the supersonic separation process. A supersonic separator was compared to a Joule-Thomson valve with TEG and the results demonstrated the high economic performance and natural gas liquids recovery of a supersonic separator [11]. The generalized radial basis function artificial neural networks were used to optimize the geometry of a supersonic separator [12]. Rajaee Shooshtari and Shahsavand developed a new theoretical approach based on mass transfer rates to calculate the liquid droplet growth in supersonic conditions for binary mixtures [13]. In our preliminary studies, a central body was incorporated in a supersonic separator with a swirling device composed of vanes and an ellipsoid [14]. The effects of swirls on natural gas flow in supersonic separators were computationally simulated with the Reynolds stress model [15]. The particle separation characteristic in a supersonic separator was calculated using the discrete particle method [16].

The mechanisms of the upstream and downstream influences are not well understood for various flow conditions from the wellhead and the back pipelines. The purpose of this study is to investigate the effects of the operating parameters on natural gas supersonic separation process, including the back pressure, inlet mass flow rates, inlet pressures and inlet temperatures. The Redlich-Kwong real gas model is employed to calculate the gas thermal properties in high pressure and low temperatures in our simulation.

## Governing equations

Natural gas can be accelerated to supersonic velocities with a Laval nozzle in a supersonic separator and, accordingly, low pressure and temperature conditions are achieved for water vapor condensation. The fluid structure of natural gas flows can be described by the conservation equations of mass, momentum and energy. To close the partial differential equations, the Shear Stress Transport (SST) [17] turbulence model was used in our simulation to solve the supersonic gas flows.

The mass equation of gas phase (continuity equation) is described as:
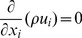
(1)where *ρ* and *u* are the gas density and velocity, respectively.

The conservation of momentum for gas phase can be written as follows:
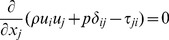
(2)where *p* is the gas pressure; *τ_ij_* is the viscous stress; *δ_ij_* is the Kronecker delta.

The energy equation for gas phase is expressed as Eq. 3.
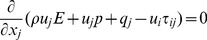
(3)where *E* is the total energy; *q_j_* is the heat flux; *t* is the time.

The turbulent kinetic energy and the specific dissipation rate equations in SST model are as follows [17], [18]:
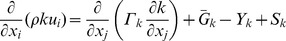
(4)

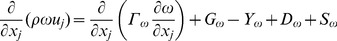
(5)where *k* is the turbulent kinetic energy, *ω* is the specific dissipation rate. 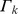 and 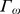 represent the effective diffusivity of k and *ω*, respectively. 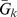 represents the generation of turbulence kinetic energy due to mean velocity gradients. *G_ω_* represents the generation of the specific dissipation rate, *ω*. *Y_k_* and *Y_ω_* represent the dissipation of *k* and *ω* due to turbulence. *D_ω_* represents the cross-diffusion term. *S_k_* and *S_ω_* are user-defined source terms.

An equation of state must be developed to calculate the physical property of fluids in supersonic flows. In this simulation, the Redlich-Kwong real gas equation of state model [19] was employed to predict gas dynamic parameters, described in Eq. (6).
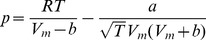
(6)where *p* is the gas pressure, *R* is the gas constant, *T* is temperature, *V_m_* is the molar volume (*V*/*n*), *a* is a constant that corrects for attractive potential of molecules, and *b* is a constant that corrects for volume.

The constants *a* and *b* are different depending on which gas is being analyzed. They can be calculated from the critical point data of the gas:
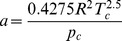
(7)

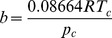
(8)where *T_c_* and *p_c_* are the temperature and pressure at the critical point, respectively.

For the multi-component mixtures, such as natural gas, mixing laws are utilized to calculate the parameters *a* and *b*. The Van Der Waals mixing rules [20], [21] were applied to obtain the parameters for the mixtures from those pure components. The mathematical expressions of this mixing rule can be written,
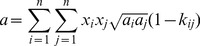
(9)

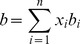
(10)where *x* is molar fraction; *n* is the total number of the gas components; *k_ij_* is the binary interaction parameter between components *i* and *j*.

## Mathematical modelling

### Computational domain and boundary conditions

**Figure 1:**
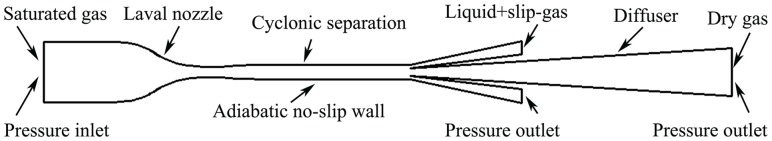
Schematic diagram of a supersonic separator.

A Laval nozzle is a key part of a supersonic separator to generate supersonic flows for the condensation and separation of natural gas. Thus, the nozzle needs to be designed specifically, as shown in Figure 1. The cubic polynomial equation was employed to calculate the converging contour of the nozzle, as shown in Eq. (11), while the Foelsch's analytical calculation method was used to design the diverging part of the nozzle [22]. This design of the converging part will accelerate the gas flow uniformly to achieve the sound speed in the throat area. The critical cross-section area is 0.0002378 m^2^. The nozzle entrance and exit areas are 0.007854 m^2^ and 0.0004460 m^2^, respectively. In addition, a straight tube with the length of 100 mm was connected to the nozzle upstream and diffuser downstream, respectively.
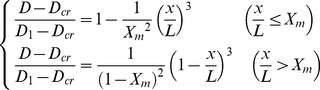
(11)where *D*_1_, *D_cr_* and *L* are the inlet diameter, the throat diameter and the convergent length, respectively. *X_m_* = 0.45. *x* is the distance between arbitrary cross section and the inlet, and *D* is the convergent diameter at arbitrary cross section of *x*.

A structured grid was generated for the supersonic separator while a finer grid scheme in the boundary layer was employed in Laval nozzle and supersonic channel. The grid independence was tested before we carried out the simulation. Boundary conditions played a significant role in a numerical simulation. In our case related to a supersonic separator, the pressure boundary conditions were assigned for the inlet and outlet of the supersonic separator, respectively, according to the flow characteristics of the supersonic compressible fluid,. No-slip and adiabatic boundary conditions were specified for the walls. The turbulent kinetic energy and turbulent dissipation rate were employed as the turbulence parameters.

### Computational methods

The finite volume methods were used to discretize the partial differential equations of the supersonic gas flows. The pressure based implicit solver was employed to solve the governing equations. The SIMPLE algorithm [23] was applied to couple the velocity field and pressure. The standard pressure scheme was adopted to interpolate the pressure values on the surface of the control volume. The second-order upwind scheme was used for other variables, such as density, momentum, turbulence kinetic energy, turbulence dissipation rate.

### Validation

**Figure 2:**
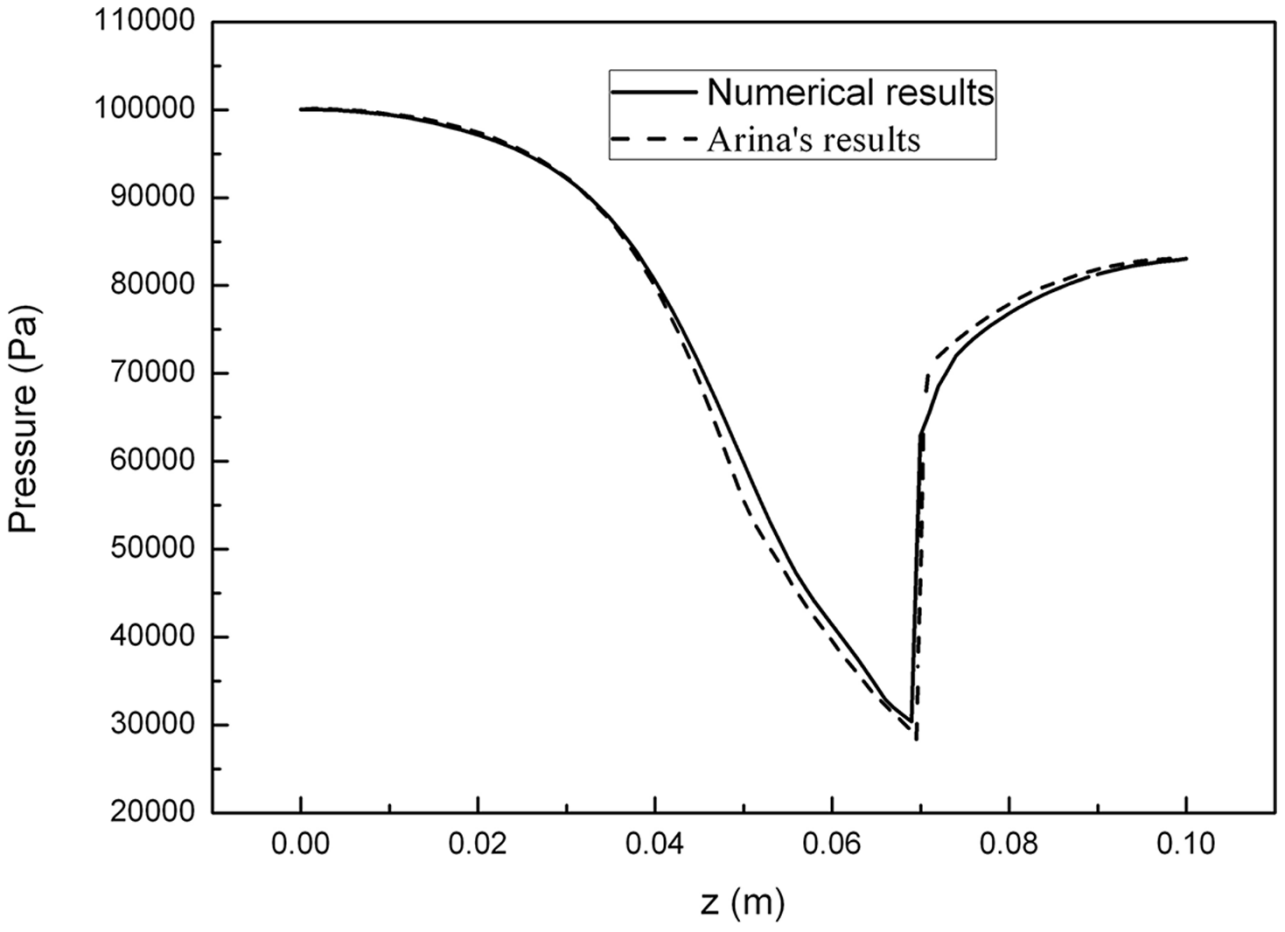
Pressure profile for nozzle flow.

For the validation of our computational methods in supersonic flows, it was validated with Arina's results before we applied it to our designed supersonic separator [24], [25]. Figure 2 depicts the pressure profiles in a Laval nozzle with the numerical results and Arina's work. It could be seen that the same flow behavior was obtained and the shock wave position was accurately captured by our simulation method. Therefore, the numerical results agree with Arina's results well. It was demonstrated that our developed model could be used in the prediction of the supersonic flow for natural gas dehydration.

## Results and Discussion

### Effect of back pressure

The flow characteristics of natural gas were numerically simulated in the supersonic separation process. The multi-components gas mixture in Baimiao gas well of Zhongyuan Oil Field was selected for the calculation. The composition of natural gas in mole fraction is shown in Table 1.

**Table 1:** Mole composition of natural gas.

Natural gas composition	Mole fraction (%)
CH_4_	91.36
C_2_H_6_	3.63
C_3_H_8_	1.44
i-C_4_H_10_	0.26
n-C_4_H_10_	0.46
i-C_5_H_12_	0.17
n-C_5_H_12_	0.16
H_2_O	0.03
CO_2_	0.45
N_2_	2.04

**Figure 3:**
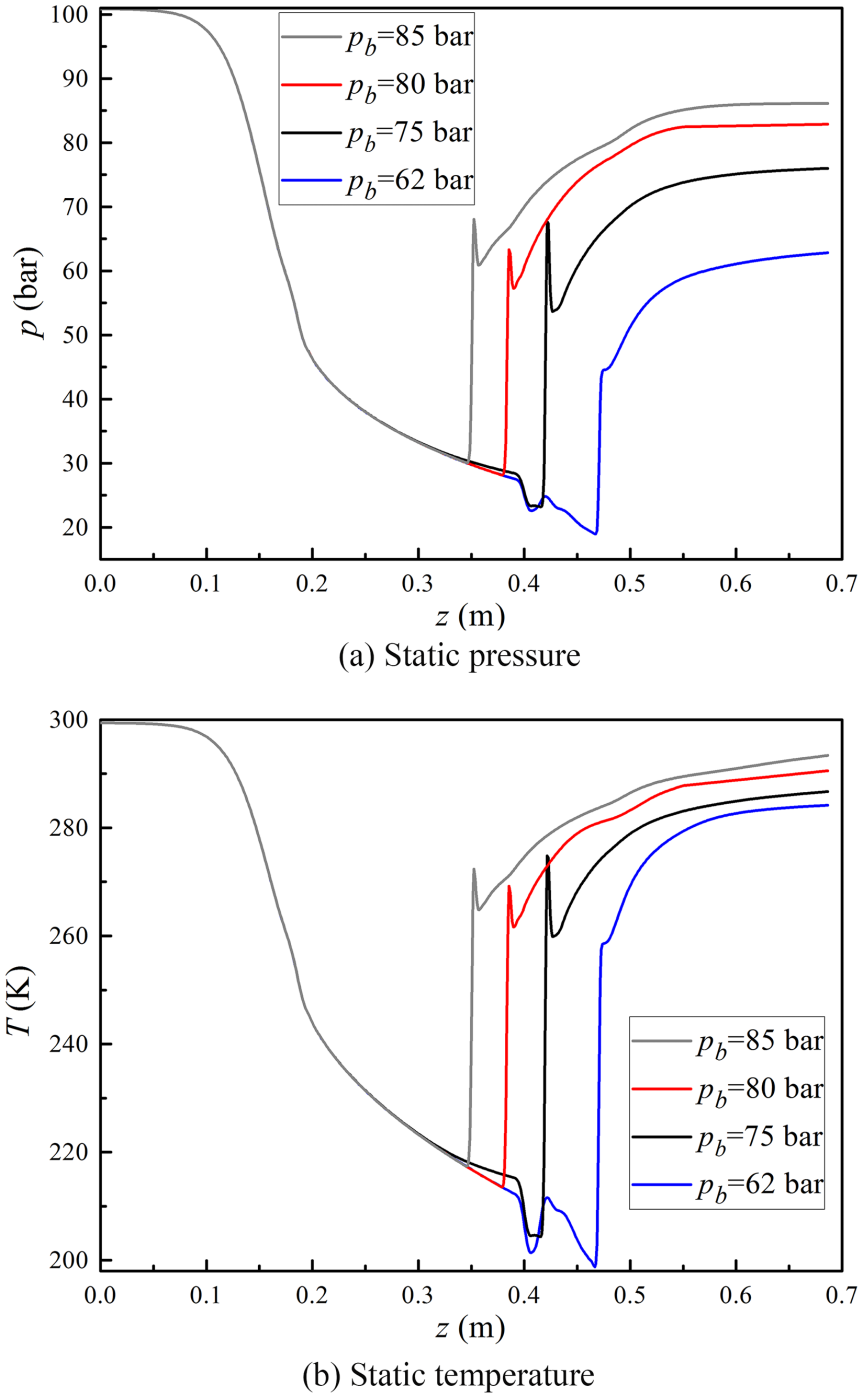
Effect of back pressure on natural gas dynamic parameters.

The incoming flow parameters are fixed when we study the effect of the back pressures on the supersonic separation process. The detailed initial conditions for the back pressure simulation are shown in Table 2. Figure 3 presents the static pressure and static temperature profiles along the flow direction in the conditions of different back pressures. The shock wave position moves into the nozzle from the diffuser with the rise of the back pressure. The shock wave will stay in the diffuser while the back pressure is about less than 75 bar with the inlet pressure of about 100 bar. If the back pressure increases to 80 bar, the shock wave will move into the supersonic channel across the diffuser entrance. The pressure and temperature profiles exhibit several fluctuations close to the shock wave and away from it. This is induced by the interaction between the boundary layer separation and the shock boundary layer.

**Table 2:** Initial conditions for back pressure simulation.

Cases	Inlet pressure (bar)	Inlet temperature (K)	Back pressure (bar)
1	100	300	85
2	100	300	80
3	100	300	75
4	100	300	62

**Figure 4:**
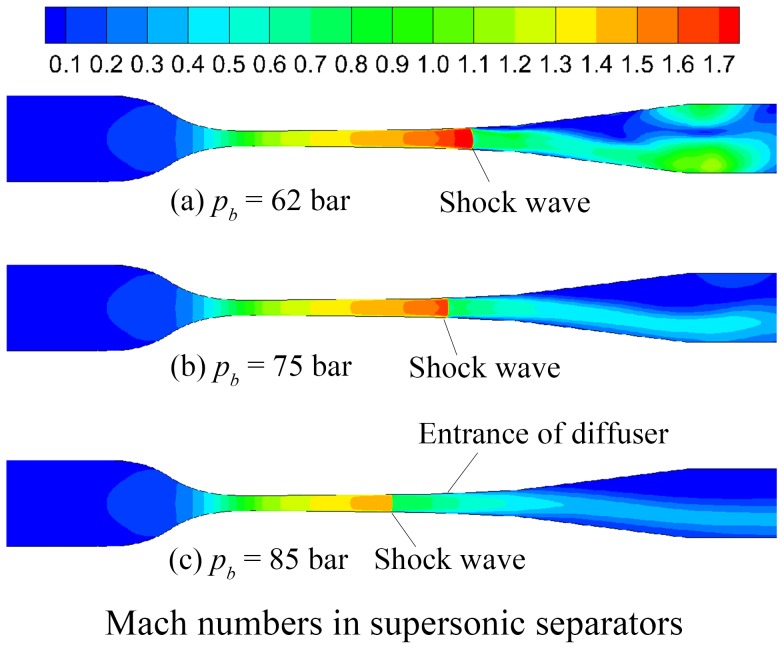
Mach numbers in supersonic separators with various back pressures.

Figure 4 depicts the contours of gas Mach numbers in the supersonic separators with various back pressures. It clearly shows the obvious differences of the shock wave position with the increasing back pressure. In this simulation case, the shock wave even goes into the nozzle diverging part when the back pressure reaches 85 bar. In this condition, the shock wave will destroy the state of the low pressure and temperature, resulting in the re-evaporation of the condensed liquids to decline the separation efficiency of the supersonic separators.

### Effect of inlet mass flow rate

**Figure 5:**
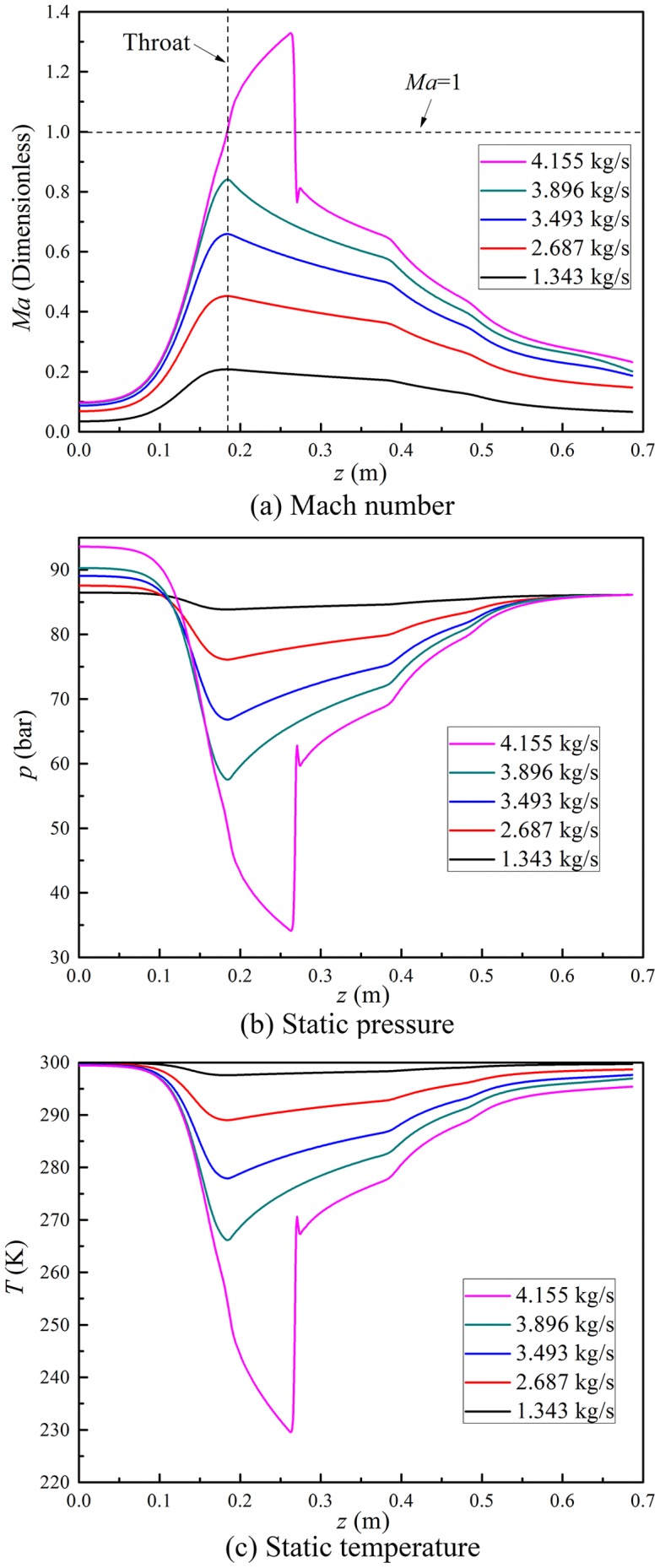
Effect of inlet mass flow rate on natural gas dynamic parameters.

A Laval nozzle is a key part in a supersonic separator, and the critical area at the nozzle throat determines the gas mass flow rate through this device. The detailed initial conditions for inlet mass flow rate simulation are shown in Table 3. Figure 5 describes the gas dynamic parameters with various inlet mass flow rate, namely, including the gas Mach number, the static pressure and static temperature. If the mass flow rate is less than the design value, the gas velocity at the nozzle throat is less than the critical value, although the converging part speeds up the gas flows. Because of the Mach number at the throat is less than unity, the gas velocity declines in the diverging part of the Laval nozzle. In this situation, the maximum velocity is obtained at the nozzle throat. That is, natural gas is the subsonic flows in the whole supersonic separator, which cannot reach the low pressure and temperature for the condensation of the water vapor. The gas Mach number rises with the increase of the inlet gas mass flow rate, resulting in the decline of the static pressure and temperature. When the inlet gas flow rate reaches the design value, the choked flow conditions will be achieved. In our simulation cases, the critical flow condition is obtained when the inlet gas mass flow rate is about 4.155 kg/s. In this condition, the natural gas flow continues to expand in the diverging part of the Laval nozzle, and the maximum Mach number is around 1.33.

**Table 3:** Initial conditions for inlet mass flow rate simulation.

Cases	Inlet mass flow rate (kg/s)	Inlet temperature (K)	Back pressure (bar)
1	1.343	300	85
2	2.687	300	85
3	3.493	300	85
4	3.896	300	85
5	4.000	300	85

**Figure 6:**
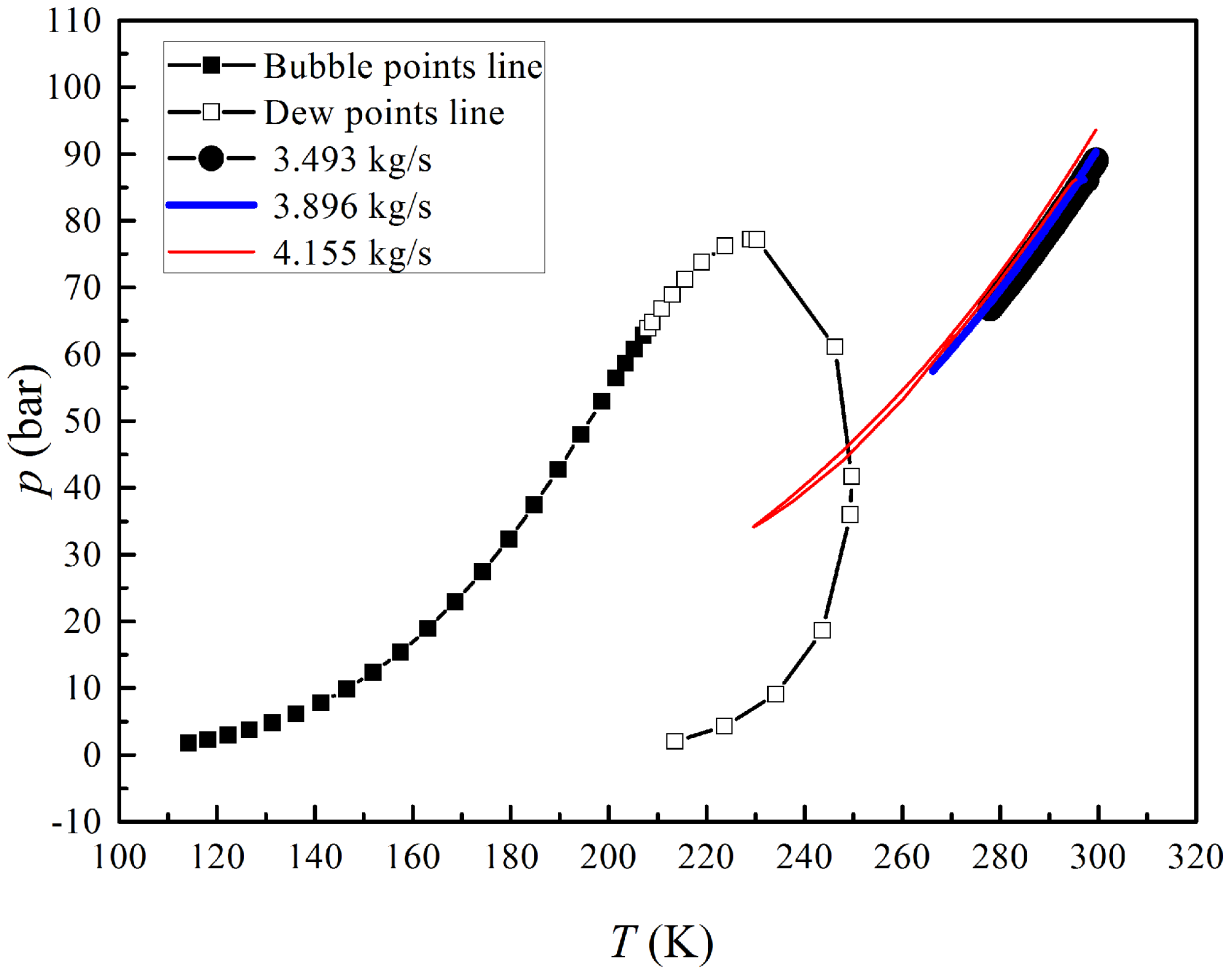
Phase envelope and pressure–temperature relationships with various inlet mass flow rates.

Figure 6 depicts the phase envelope curve and the pressure–temperature (P-T) profiles with various inlet mass flow rates. We can see that P-T profile doesn't reach the phase envelope curve because of the high pressure and temperature in the supersonic separator, when the inlet gas mass flow rate is smaller than the design value. Therefore, the water vapor can hardly be removed from natural gas when the inlet mass flow rate is less than the designed rate.

### Effect of inlet pressure

**Figure 7:**
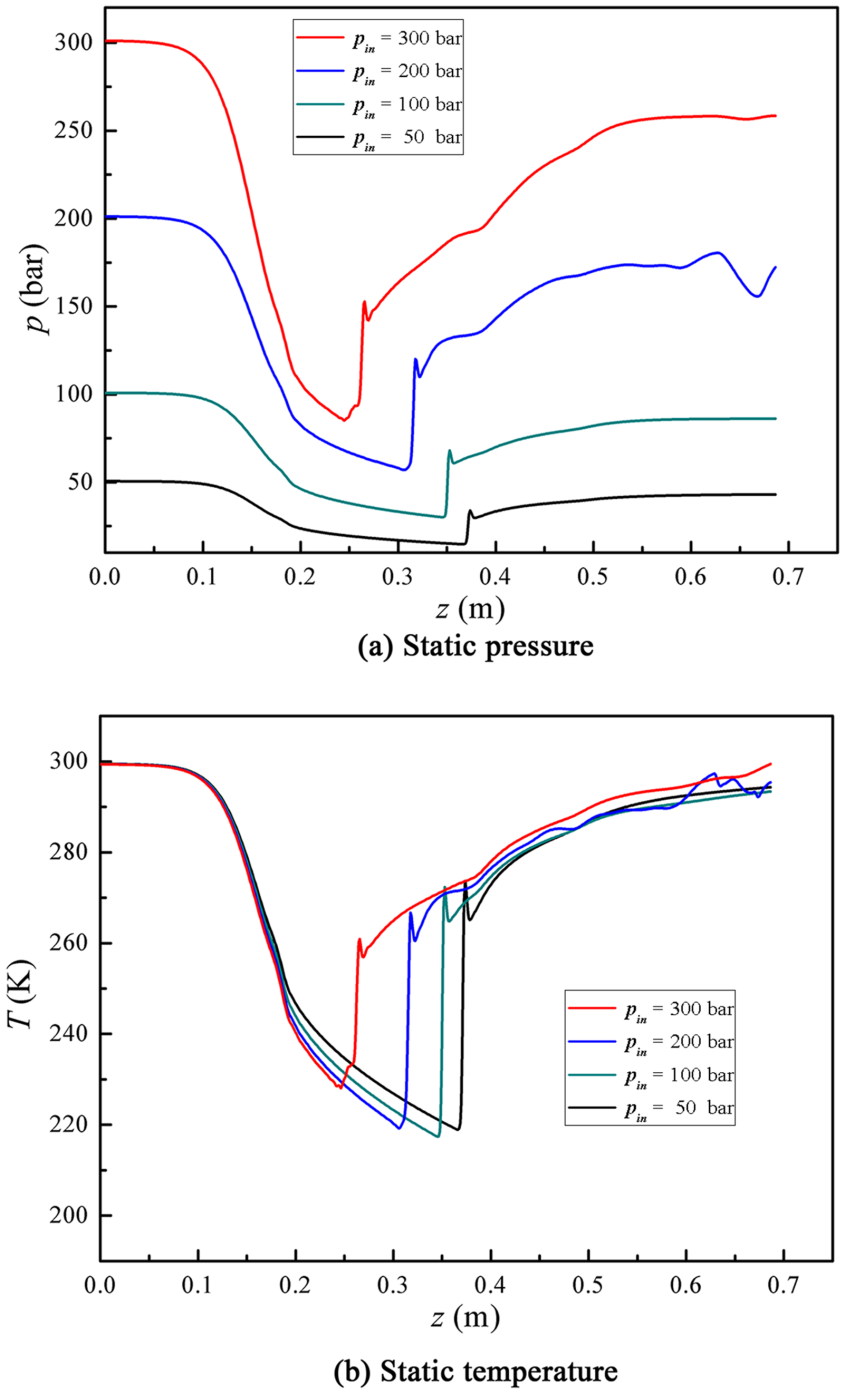
Effect of inlet pressure on natural gas dynamic parameters.

The inlet temperature is fixed and the back pressure is set to be the 85% of the inlet one, when we studied the effect of the inlet pressures on the gas dynamic parameters. The detailed initial conditions for inlet pressure simulation are shown in Table 4. The gas mass flow rate in a supersonic separator increases with the rises of the inlet pressure. It indicates that the processing capacity of a supersonic separator can be improved by increasing the inlet pressure in natural gas processing. Figure 7 presents the gas static pressure and temperature profiles along the designed supersonic separator. The shock wave position shifts forward to the nozzle with a higher inlet pressure. For example, the shock position stayed at z = 0.370 m at an inlet pressure of about 50 bar. However, the shock location goes to the upstream of the nozzle divergent part, at z = 0.263 m, when the inlet increases to 300 bar. That is, the shock wave position shifts forward by a distance of about 97 mm. This numerical simulation indicates that the pressure recovery capacity of the supersonic separator will decline in a higher inlet pressure.

**Table 4:** Initial conditions for inlet pressure simulation.

Cases	Inlet pressure (bar)	Inlet temperature (K)	Back pressure (bar)
1	50	300	42.5
2	100	300	85
3	200	300	170
4	300	300	255

**Figure 8:**
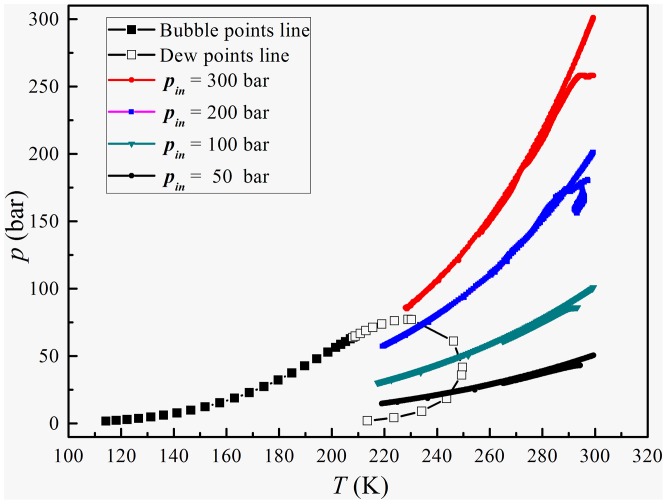
Phase envelope and pressure–temperature relationships with various inlet pressures.

Figure 8 depicts the phase envelope curve and the pressure–temperature (P-T) profiles with various inlet pressures. The P-T profile goes into the gas-liquid two phase zone, although the inlet pressure is changed, when the inlet pressure is lower than 100 Bar. In these conditions the static pressure and temperature is low enough for the condensation of the water vapor in natural gas. But if the inlet pressure exceeds 200 bar, the natural gas flow will present a supercritical fluid in the supersonic separator, which is not suitable for the gas dehydration. Therefore, we suggest that the maximum inlet pressure should be around 100 bar for natural gas dehydration using a supersonic separator.

### Effect of inlet temperature

**Figure 9:**
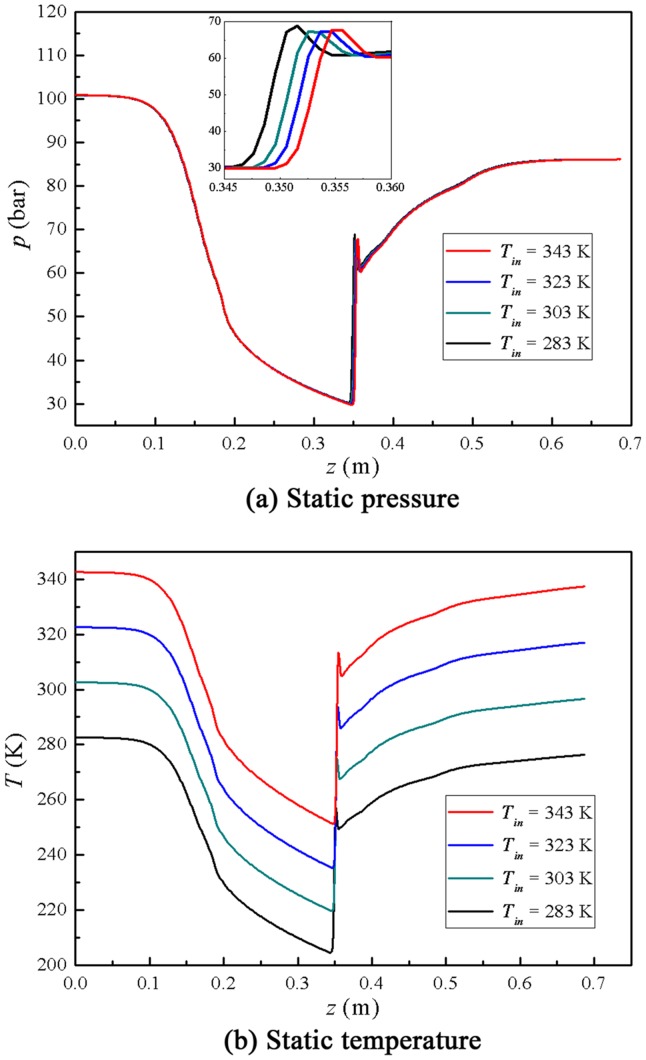
Effect of inlet temperature on natural gas dynamic parameters.

**Figure 10:**
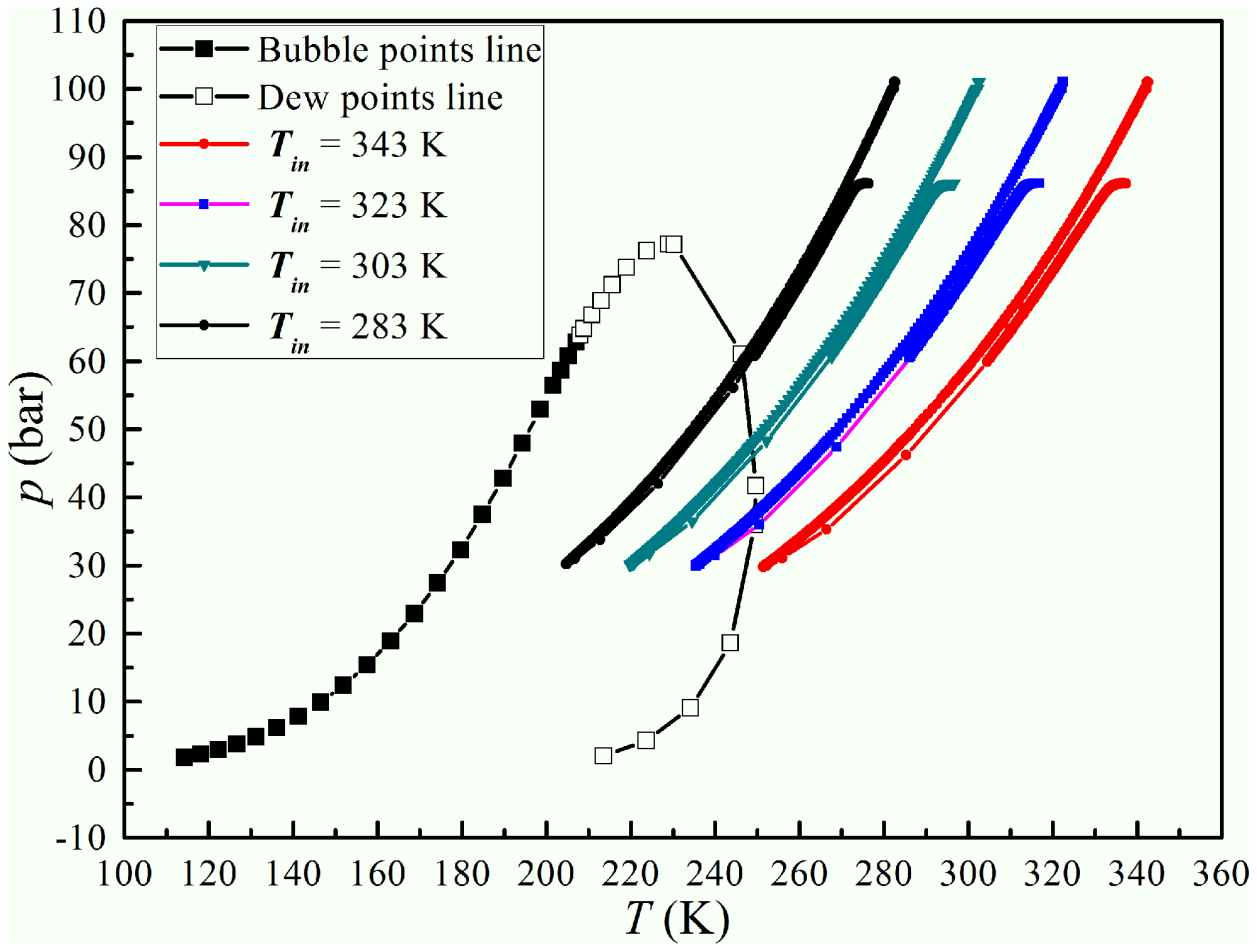
Phase envelope and pressure–temperature relationships with various inlet temperatures.

The inlet and back pressure are fixed to study the influence of the inlet temperature. The detailed initial conditions for inlet temperature simulation are shown in Table 5. The gas mass flow rate decreases with the rise of the inlet temperature in the supersonic separator. It indicates that the processing capacity of a supersonic separator can be improved by decreasing the inlet temperature in natural gas processing. It can be seen in Figure 9 that the shock position moves backward from nozzle to diffuser with the increase of the inlet temperature. However, the shock position moves just by a distance of about 5 mm with the increase of the inlet temperature from 10°C to 70°C, which is the normal temperature in natural gas processing. Hence, we can neglect the effect of the inlet temperature on the shock wave position in the supersonic separator. Figure 10 shows that the P-T profile goes further into the gas-liquid two phase zone with the decline of the inlet temperature. This is because the lower inlet temperature will cause a lower static temperature in the Laval nozzle, when the pressure ratio is fixed in the supersonic separator.

**Table 5:** Initial conditions for inlet temperature simulation.

Cases	Inlet pressure (bar)	Inlet temperature (K)	Back pressure (bar)
1	100	283	85
2	100	303	85
3	100	323	85
4	100	343	85

## Conclusion

The gas dynamic parameters in a supersonic separator were simulated using the Shear Stress Transport (SST) turbulence model and Redlich–Kwong real gas model. The effect of the inlet and outlet flow conditions on the gas dynamic parameters was analyzed in the supersonic separation process, especially on the shock wave position. The gas flow cannot be choked in the supersonic separator, when the inlet mass flow rate is less than the designed one. It results in a high pressure and temperature inside the device and the water vapor cannot be removed from natural gas. The shock wave position shifts forward to the nozzle with a higher inlet pressure. The effect of the inlet temperature on the shock wave position can be neglected when the inlet temperature increases from 10°C to 70°C. The increasing back pressure induces the shock wave position to move forward from the diffuser to Laval nozzle. The shock wave moves into the supersonic channel or Laval nozzle when the back pressure is about more than 75 bar with the inlet pressure of about 100 bar. The shock wave will destroy the state of the low pressure and temperature, resulting in the re-evaporation of the condensed liquids.
